# Protective role of Cadherin 13 in interneuron development

**DOI:** 10.1007/s00429-017-1418-y

**Published:** 2017-04-06

**Authors:** Abigail C. Killen, Melissa Barber, Joshua J. W. Paulin, Barbara Ranscht, John G. Parnavelas, William D. Andrews

**Affiliations:** 1 Department of Cell and Developmental Biology, University College London, Gower Street, London, WC1E 6BT UK; 2 Department of Neuroscience, Physiology and Pharmacology, University College London, London, UK; 3 Sanford-Burnham Prebys Medical Discovery Institute, La Jolla, CA USA

**Keywords:** Cell migration, Cadherin, Interneurons, Apoptosis

## Abstract

Cortical interneurons are generated in the ganglionic eminences and migrate through the ventral and dorsal telencephalon before finding their final positions within the cortical plate. During early stages of migration, these cells are present in two well-defined streams within the developing cortex. In an attempt to identify candidate genes which may play a role in interneuron stream specification, we previously carried out a microarray analysis which identified a number of cadherin receptors that were differentially expressed in these streams, including Cadherin-13 (Cdh13). Expression analysis confirmed Cdh13 to be present in the preplate layer at E13.5 and, later in development, in some cortical interneurons and pyramidal cells. Analysis of Cdh13 knockout mice at E18.5, but not at E15.5, showed a reduction in the number of interneurons and late born pyramidal neurons and a concomitant increase in apoptotic cells in the cortex. These observations were confirmed in dissociated cell cultures using overexpression and short interfering RNAs (siRNAs) constructs and dominant negative inhibitory proteins. Our findings identified a novel protective role for Cdh13 in cortical neuron development.

## Introduction

Cortical interneurons originate in the ganglionic eminences (GEs) in the ventral telencephalon and migrate in tangentially oriented streams to enter the cerebral cortex (Marin and Rubenstein 2003; Metin et al. 2006). Here they assemble into functional circuits with their pyramidal counterparts and contribute to a precise balance of synaptic excitation and inhibition. A plethora of factors have been identified as regulators of interneuron migration (Faux et al. 2012; Guo and Anton 2014; Marin 2013), but little is known about those that determine the choice of stream they use to enter the cortex. In order to identify the genes involved in migratory stream specification, we compared the gene expression profiles of cells in the preplate layer (PPL) with those of cells migrating through the intermediate zone (IZ) during early corticogenesis. Our analysis identified several cadherin family members that showed differential expression, including Cdh13 (also known as T-cadherin) which was present only in the PPL (Antypa et al. 2011).

Cdh13 is an atypical member of the cadherin family, devoid of a transmembrane domain and anchored to the exterior surface of the plasma membrane via a glycosylphosphatidylinositol (GPI) anchor (Ranscht and Dours-Zimmermann 1991). It is thought to affect cellular behaviour largely through its binding partners and signalling properties (Philippova et al. 2003, 2009). In the human central nervous system, CDH13 is found in a variety of cell types, including cortical pyramidal and non-pyramidal neurons and astrocytes, and is expressed at lower levels in the developing brain than in the adult (Takeuchi et al. 2000).

Using an RNAi-based gene silencing approach, Cdh13 has been shown to play a role in glutamatergic and/or GABAergic synapse development in vitro (Paradis et al. 2007) and in the aggregation and neurite outgrowth of cultured motor neurons (Ciatto et al. 2010). Recently, CDH13 has been associated with hyperactive/impulse symptoms in attention deficit hyperactive disorder (ADHD) (Salatino-Oliveira et al. 2015). Two other recent studies have shed light on the mechanism by which suppression of Cdh13 function may contribute to ADHD. Firstly, suppression of Cdh13 function in lower layer cortical neurons by RNAi leads to aberrant axonal projections (Hayano et al. 2014) and, secondly, Cdh13^−/−^ mice display deficits in learning and memory due to alterations in neuron function in the hippocampus, similar to that observed in ADHD patients (Rivero et al. 2015).

Here we wanted to explore further the roles of Cdh13 function in cortical development. We observed the presence of Cdh13 throughout the whole period of corticogenesis, with particularly strong expression detected in pyramidal neurons and interneurons at later stages. Analysis of Cdh13^−/−^ mice revealed decreased number of both neuron types at E18.5 and a concomitant increase in the level of apoptosis, suggesting that Cdh13 plays a critical protective role in cell survival at late stages of cortical development.

## Materials and methods

### Animals

All experimental procedures were performed in accordance with the UK Animals (Scientific Procedures) Act 1986 and institutional guidelines. Wild-type animals were C57/bl6J mice obtained from Charles River Ltd. *Cdh13* ^−/−^ knockout (KO) mice and *GAD67-GFP* ^*neo*/−^ mice were generated as described previously (Hebbard et al. 2008; Tamamaki et al. 2003). The day the vaginal plug was found was considered as embryonic day (E) 0.5. Animals of both sexes were used in our experiments.

### In situ hybridization

For in situ hybridization and immunohistochemistry, embryonic brains were dissected in phosphate-buffered saline (PBS) and fixed in 4% paraformaldehyde (PFA), made in PBS, for 4–8 h at room temperature (RT). Following fixation, embryonic brains were cryoprotected in 30% sucrose in Diethyl Pyrocarbonate (DEPC)-treated PBS, embedded and frozen in a mixture of 15% sucrose/50% Tissue-Tek OCT (Sakura Finetek) and sectioned in the coronal plane at 20 µm using a Cryostat (Bright Instruments). Sections were dried at RT for 2 h, before overnight incubation at 65 °C in hybridization buffer [1X DEPC-treated ‘Salts’ (200 mM NaCl, 5 mM EDTA, 10 mM Tris pH 7.5, 5 mM NaH_2_PO_4_.2H_2_O, 5 mM Na_2_HPO_4_; Merck KGaA); 50% deionized formamide (Ambion); 0.1 mg/ml RNAse-free yeast tRNA (Life Technologies); 1x Denhardt’s (RNase/DNase free; Life Technologies); 10% dextran-sulphate (Merck KGaA)] containing 100–500 ng/ml DIG labelled-RNA probes. Probes were generated by linearization of plasmids with appropriate enzymes and reverse transcription polymerases to obtain antisense probes. Probes used were: *Cdh13* (provided by B.R.) and GAD67 (kindly provided by Dr. Brian Condie, University of Georgia, Georgia, USA). Following hybridization, sections were washed 3 times in 50% formamide 1XSSC (Ambion) and 0.1% Tween-20 (Merck KGaA) at 65 °C and 2 times at RT in 1XMABT (20 mM Maleic acid, 30 mM NaCl, 0.1% Tween-20; Merck KGaA) before incubating in blocking solution [2% blocking reagent (Roche), 10% normal goat serum (Vector Laboratories) in MABT] followed by overnight incubation in alkaline phosphatase-conjugated anti-DIG antibody (1:1500; Roche). Nitroblue tetrazolium chloride/5-bromo-4-chloro-3-indolyl phosphate (Roche) diluted 1:1000 in MABT with 5% polyvinyl alcohol (VWR International Ltd) was used for colorimetric detection for 6 h. Fast Red (Roche) was used for fluorescence colour detection of probes by incubation in 100 mM Tris (pH 8.0) and 400 mM NaCl containing Fast Red at 37 °C for approximately 2 h. Fluorescent in situ hybridization was followed by immunohistochemical detection of GFP as described below. Sections were mounted with Glycergel Mounting Medium (Dako).

### Immunohistochemistry

Embryonic brain sections were washed in PBS, blocked in a solution of 5% normal goat serum (Merck KGaA) (v/v) containing 0.1% Triton X-100 (v/v) (Merck KGaA) in PBS at RT for 2 h. They were first incubated in primary antibodies at RT for 2 h and, then, at 4 °C overnight. The following antibodies were used: mouse monoclonal Nestin (1:100, DSHB) and 5-Bromodeoxyuridine (BrdU; 1:1000, Progen), rat monoclonal anti-Ctip2 (1:500, Abcam), chicken polyclonal raised against GFP (1:500, Aves Laboratories), rabbit polyclonal raised against calbindin (CB-28; 1:3000, Swant), cleaved caspase-3 (CC3; 1:250, Cell Signalling Technology), Cux1 (1:100, Santa Cruz Biotechnology), Cdh13 (1:500, Millipore), L1 (L1; 1:1000, Millipore) or phospho-histone H-3 (PH-3; 1:1000, Millipore). Following incubation in primary antibodies, sections were washed in PBS, incubated in biotinylated anti-species secondary antibodies (1:250; Vector Laboratories) for 2 h and processed using conventional immunohistochemistry protocols described previously (Andrews et al. 2008).

### GAD67 interneuron and Ctip2/Cux1 pyramidal neuron counts

In Cdh13 knockout tissues at E15.5 and E18.5, a 300 μm segment was measured along the ventricular surface of the cortex next to the cortico-striatal junction. A rectangle was then drawn to incorporate the entire thickness of the cortex within the 300 μm, and the number of stained cells in that box was counted. For interneurons, the number of GAD67+ cells in each layer was recorded as well as the total number of neurons. For Ctip- and Cux1-labelled pyramidal cells, counts were only made in their specific layers within the boxed region.

### Quantification of PH-3-positive cells

All PH-3-positive cells present along the entire ventricular zone/subventricular zone (VZ/SVZ), from the cortico-striatal junction to the cortical hem (CH), throughout the rostral-caudal extent of the cortex in E15.5 embryonic coronal sections were included in all measurements (minimum of 8 sections from each of 4 animals for each genotype). The extent of the layers was determined by methyl green counterstaining (Vector Laboratories). Quantification of apical progenitors lining the VZ was presented as PH-3-labelled cells per mm. Basal progenitors in the SVZ were presented as PH-3-labelled cells per 10^4^ per µm^2^. Basal progenitors here were defined as any cell more than three cells width away from the ventricle surface.

### Caspase apoptotic cell counts

Sections taken through the brains of *Cdh13* ^+/+^ and *Cdh13* ^−/−^ embryos at E15.5–E18.5 (*n* = 3 for each genotype) were immunostained for CC3. A 300-μm-wide rectangle was then drawn to incorporate the entire thickness of the cortex and the number of CC3+ cells in the CP within the box was counted. CC3+ blood vessels were excluded from the counts.

### Plasmids and RNAi constructs

For overexpression experiments, we first generated mouse cDNA from E13.5 mouse forebrain mRNA obtained using RNAeasy kit (Qiagen) and Superscript III cDNA synthesis kit (Life Technologies). Mouse *Cdh13* cDNA was produced by PCR amplified using *Pfu* polymerase (Promega) [Forward (*NheI*), GCTAGCGATGCAGCCGAGAACTCCGCTC; Reverse (*HindIII*), CCCAAGCTTCAGAC-CTGACAATAAGC], digested with *NheI* and *HindIII* and subcloned into the pCDNA3.1(−) expression vector (Promega). For RNAi experiments, we designed three different oligonucleotides, targeting specific regions of mouse *Cdh13* cDNA [S1 specifically recognizes nucleotides 278–299; S2, nucleotides 455–476; and S3, nucleotides 1364–1385] (GenBank accession number NM_019707). Three oligonucleotides targeting the corresponding regions of mouse *Cdh13* cDNA were used in dissociated cell culture experiments.

Annealed oligonucleotides were cloned in the GeneClip™ U1 Hairpin-hMGFP vector according to the manufacturer’s instructions (Promega). As controls, we used short interfering RNAs (siRNAs) targeting the same regions, but containing three point mutations and, thus, not affecting the stability of *Cdh13* mRNA. The efficiency of the three different shRNAs in targeting *Cdh13* mRNA was determined by co-transfection mouse *Cdh13* cDNA and the different shRNAs at a ratio 1:3, using Lipofectamine 2000 (Life Technologies) into COS-7 cells. After 48 h, mRNA and protein were harvested and level of knockdown was determined. mRNA fold change is defined as level of test conditioned divided by control (S1m).

### QPCR

cDNA from transfected cells and E18.5 interneuron cultures was analysed by qPCR. The qPCR reaction was performed with SYBR Green reagent (Merck KGaA) on a CFX96™ Real-Time PCR Detector system (Bio-Rad) in accordance with manufacturer’s instructions. Primers for quantitative real-time PCR (QPCR) were designed by Merck KGaA and were as follows: Akt (forward, ATGAACGACGTAGCCATTGTG; reverse, TTGTAGCCAATAAAGGTGCCAT); Bcl2 (forward: GTCGCTACCGTCGTGACTTC; reverse: CAGACATGCACCTACCCAGC), *Casp2* (forward, TACTCCCACCGTTGAGCTGT; reverse, CCGTAGCATCTGTGGATAGGC); *Cdh13* (forward, ACCAGCCTGTCCTAAACTTGA; reverse, GCTCCAGAGTTCGGGTGTG); *Gapdh* (forward, ATGACATCAAGAAGGTGGTG; reverse, CATACCAGGAAATGAGCTTG); *Pipa* (forward, GAGCTGTTTGCAGACAAAGTTC; reverse, CCCTGGCACATGAATCCTGG); *Pi3k* (forward, ACACCACGGTTTGGACTATGG; reverse, GGCTACAGTAGTGGGCTTGG). *Gapdh* and *Pipa* were used for endogenous reference gene controls. Each primer set amplified a single PCR product of predicted size as determined by melt-curve analysis following PCR and by agarose gel electrophoresis and had approximately equal amplification efficiencies when validated with a serial dilution of representative cDNA. Each qPCR was performed in triplicate, and relative quantification was determined according to the DDc(t) method (Faux et al. 2010; Livak and Schmittgen 2001).

### Western blots

COS7, GN11 cells, or interneurons were lysed in lysis buffer (50 mM Tris–HCl, pH 7.4, 150 mM NaCl, 1% Nonidet P40). The lysate was incubated at 4 °C for 30 min and centrifuged for 5 min. Cell lysates were processed for conventional SDS–PAGE and membrane transfer. Membranes were incubated with the following rabbit polyclonal antibodies: Phospho^ser473^-Akt (1:1,000; Cell Signalling Technology), Total Akt (1:1000; Cell Signalling Technology), β-catenin (1:1000; Abcam), Bcl2 (1:1000; Miliore), Cdh13 (1:1000; Miliore), Gapdh (1:500; Merck KGaA), Total Gsk3β (1:1000; Cell Signalling Technology), Phospho^ser9^-Gsk3β (1:1,000; Cell Signalling Technology) in 5% BSA-TBST, washed several times with TBST and incubated with a horseradish peroxidase conjugated secondary antibody (1:5000; Vector Laboratories). After intensive washing, the proteins were visualized with ECL detection reagent (GE Healthcare).

### Dissociated MGE cell cultures

Dissociated cell cultures were prepared from embryonic mice as described previously (Cavanagh et al. 1997). Briefly, MGEs were dissected out in cold artificial cerebrospinal fluid (ACSF) under a stereomicroscope. They were incubated in Neurobasal medium (Thermo Fisher Scientific) containing 0.05% trypsin (Merck KGaA) and 100 µg/ml DNase I (Roche) at 37 °C for 15 min. Trypsinization was quenched with Neurobasal medium containing 10% of FBS (Thermo Fisher Scientific) at 37 °C for 5 min. MGEs were then triturated by pipetting until no cellular aggregates were visible. The homogenous cell suspensions were subsequently pelleted by centrifugation at 1000×*g* for 3 min. Cells were re-suspended in dissociation media (DM) [DMEM/F12 culture media containing B27 supplement, 100 µg/ml penicillin/streptomycin and 2 mM L-glutamine (Thermo Fisher Scientific)], and 100,000 cells were seeded onto 13 mm coverslips coated previously with 10 µg/ml poly-l-lysine and 10 µg/ml laminin (Merck KGaA) and incubated in a humidified incubator at 37 °C. The culture medium was changed the next day.

For cell signalling experiments, E15.5 MGE cell cultures were prepared, and the next day fresh DM was added, containing either 2 µg/ml human IgG or 2 µg/ml Cdh13-Fc, and left for 2 DIV. Cells were then processed either for immunohistochemistry, Western blotting or qPCR.

### Chemotaxis assays

Chemotaxis assays were performed using a 48-well Boyden’s chamber (Neuro Probe) as described previously (Hernandez-Miranda et al. 2011). Briefly, 27 µl of DM containing 1% FBS were placed into the lower compartment of the chamber. Dissociated MGE or GN11 cells were re-suspended in serum-free medium (10^5^ cells/50 µl) and placed in wells of the upper compartment of the chamber in the presence of anti-Cdh13 antibody (2 µg/ml, R&D Systems) or rabbit serum (Merck KGaA). These were separated from the lower chamber by a polycarbonate porous membrane (8 µm pores), pre-coated with gelatin (0.2 mg/ml) for GN11 cells and laminin (10 µg/ml), plus either 1 µg/ml human IgG (Merck KGaA) or 1 µg/ml Cdh13-Fc (R&D Systems). For GN11 cells, the chamber was kept in an incubator at 37 °C for 4 h (GN11 cells) or overnight (MGE cells). After incubation, the migrated cells that adhered to the underside of the membrane were fixed and stained using the Diff-Quik kit (Reagena). For quantitative analysis, the membranes were observed using an Olympus light microscope with a 20× objective adapted with a 500 × 500 µm grid. Four random fields of stained cells were counted for each well, and the mean number of migrating cells per square millimetre for each experimental condition was estimated.

### Proliferation/apoptosis experiments

48 h after transfection with overexpression and knockdown constructs, primary cells were washed, fixed with 4% PFA, immunostained for PH-3 and nestin to identify progenitors, and EGFP to identify transfected cells. For analysis of apoptosis in interneuron cultures, CC3 and CB immunohistochemistry was carried out. Cell counts were made using a ×40 objective lens in 9 fields of view for each sample. At least three samples were evaluated for each time point and treatment. For overexpression constructs, the controls were cells that had been transfected with empty pcDNA3.1 (Life Technologies) vector. For knockdown constructs, the controls were cells that had been transfected with mutated siRNA constructs in the same pGeneClip™ hMGFP vector. The percentage of proliferating cells was determined using the following equation: total number of EGFP-nestin double-labelled cells divided by the number of PH3+- EGFP-nestin triple positive cells multiplied by 100. To calculate the percentage of apoptotic interneurons in the cultures, we used the following equation: total number of EGFP+-CB+ cells divided by the number of CC3+-EGFP+-CB+ cells multiplied by 100.

### Digital image acquisition and processing

Optical and fluorescent images were collected using Leica Microsystems light- or confocal microscopes. Images were reconstructed and digitized with Photoshop CS2 software (Adobe Systems).

### Statistics

All data are reported as mean number and SEM. Statistical analysis was performed with GraphPad 3 (GraphPad Software). A one-way ANOVA was used to evaluate the fit of the data to a normal distribution and, then, Student’s *t* test was used to evaluate paired comparisons. Significant differences were considered when *p* < 0.05.

## Results

### Expression of Cdh13 in the developing forebrain

Little is known about the expression of Cdh13 in the embryonic rodent telencephalon. Earlier expression studies were performed in other species and, for the most part, at later ages than at key stages of cortical interneuron development (Matsunaga et al. 2013; Ranscht and Dours-Zimmermann 1991; Takeuchi et al. 2000). Thus, we undertook an extensive analysis of Cdh13 expression at early (E13.5), middle (E15.5) and late (E18.5) stages of mouse embryonic cortical development using in situ hybridization and immunohistochemistry.

**Fig. 1:**
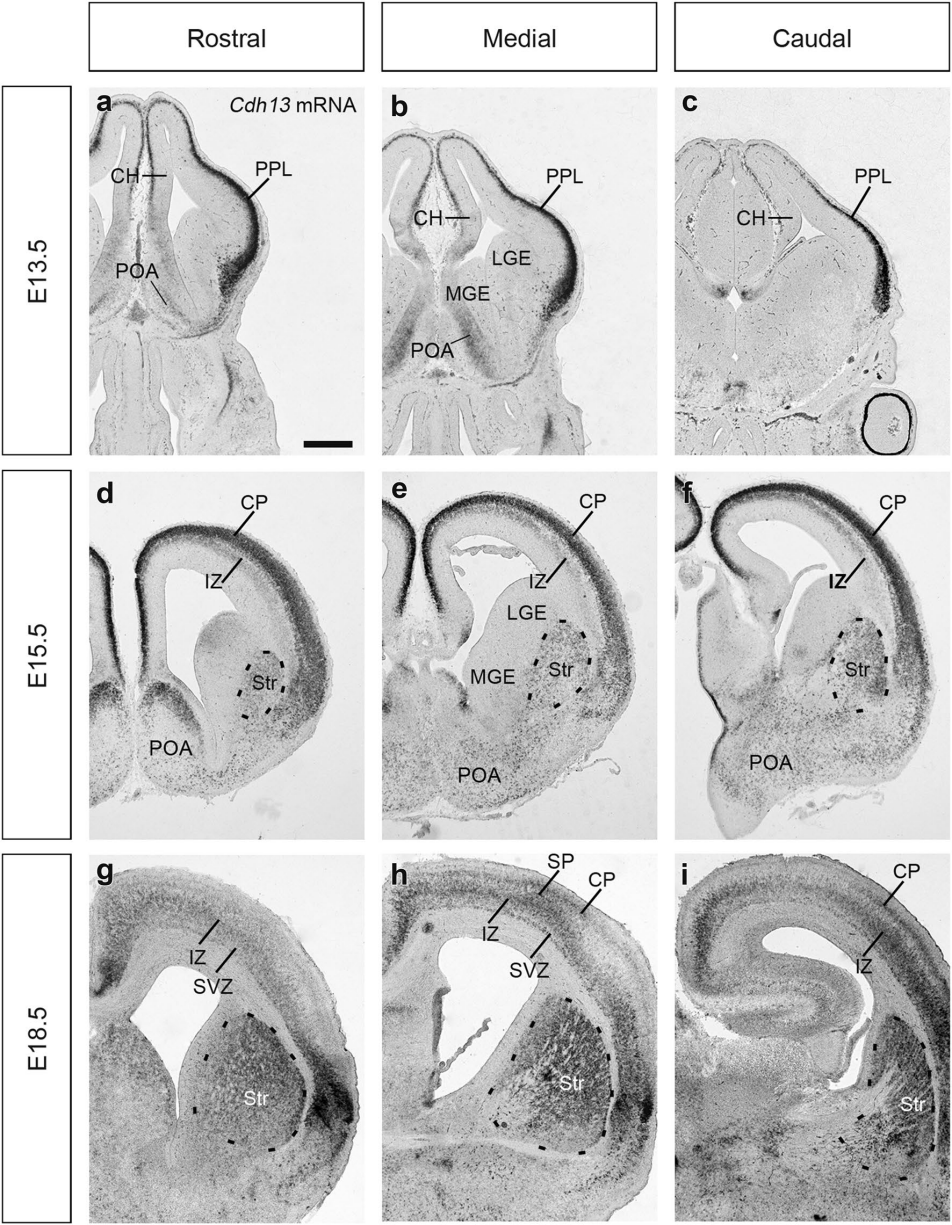
In situ hybridization for *Cdh13* mRNA in the developing forebrain. Coronal sections through the developing mouse forebrain at E13.5 (**a**–**c**), E15.5 (**d**–**f**) and E18.5 (**g**–**i**) showing localization of *Cdh13* expression at rostral (**a, d, g**), medial (**b, e, h**) and caudal (**c, f, i**) levels. *Scale bar* in **a**, 500 μm, applicable to all images. *CH* cortical hem, *CP* cortical plate, *IZ* intermediate zone, *LGE* lateral ganglionic eminence, *MGE* medial ganglionic eminence, *POA* preoptic area, *PPL* preplate layer, *Str* striatum, *SP* subplate, *SVZ* subventricular zone

At E13.5, *Cdh13* mRNA expression shows a gradient within the PPL being highest at the level of the cortico-striatal junction and lowest in the hippocampus (Fig. 1a–c); this is in agreement with our previous microarray study (Antypa et al. 2011). In the basal telencephalon, *Cdh13* mRNA is expressed weakly in the preoptic area (POA). At E15.5, it is expressed strongly in the cortical plate (CP) with a weaker, secondary band alongside the upper IZ that increases in strength from the cortico-striatal junction (Fig. 1d–f). Its expression in the basal telencephalon at this stage appears to have expanded too, extending further ventrally, particularly in the region of the lateral ganglionic eminence (LGE) where it now encompasses the developing striatum (Str) (Fig. 1d–f).

By E18.5, expression in the cortex becomes much broader, extending from the CP, across the IZ and into the upper SVZ. In addition, within this band, there are stripes of strong expression in the SVZ, IZ and low-mid CP/subplate (SP) (Fig. 1g–i). Expression is particularly strong in the Str in the basal telencephalon. Our finding that Cdh13 expression increases as development proceeds in the cortex concurs with the recent study of Rivero et al. (2015).

**Fig. 2:**
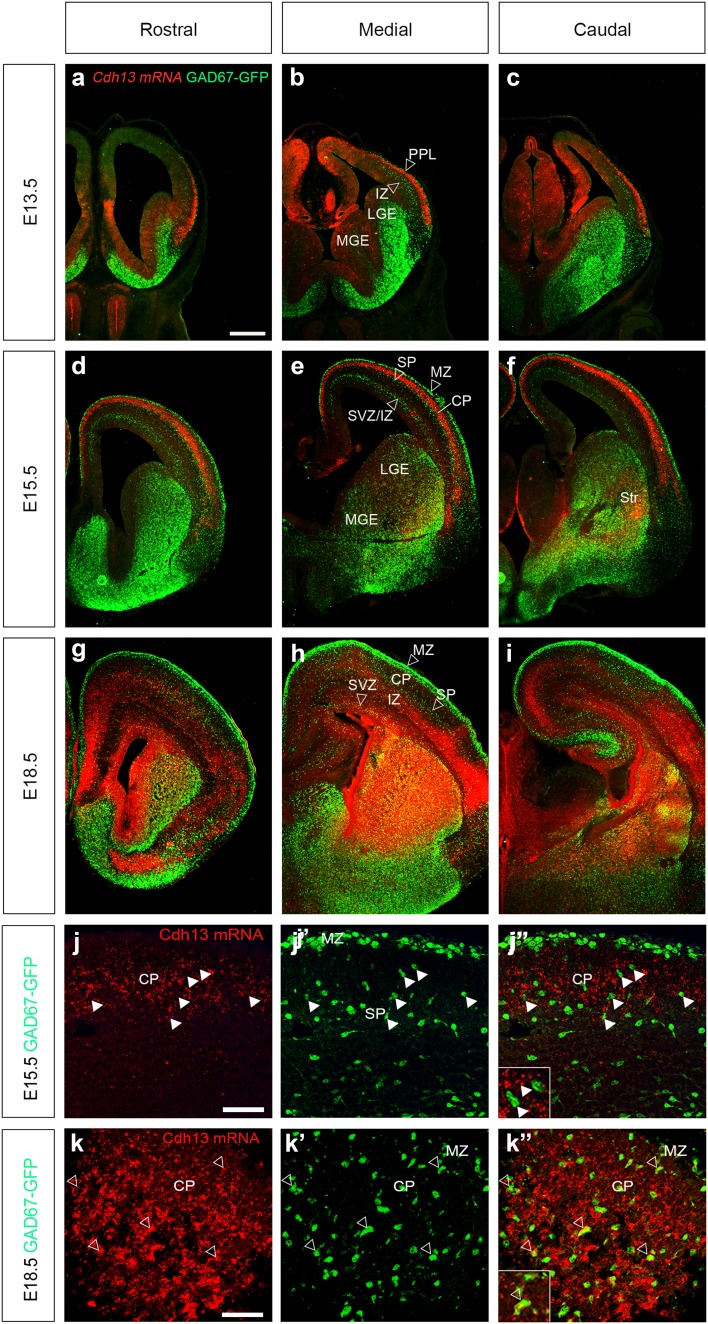
Fluorescence in situ hybridization for *Cdh13* mRNA in the developing forebrain. Coronal sections from GAD67-GFP mouse forebrains at E13.5 (**a**–**c**), E15.5 (**d**–**f**) and E18.5 (**g**–**i**) showing *Cdh13* expression (*red*) at rostral (**a, d, g**), medial (**b, e, h**) and caudal (**c, f, i**) levels. *Hollow arrowheads* indicate the positions of migratory streams of cortical interneurons. **j**–**k**″) Higher power images at E15.5 (**j, j**″) and E18.5 (**k, k**″); arrowheads indicate examples of interneurons that express *Cdh13* mRNA (see *insets*). *Scale bar* in **a** 500 μm (**a**–**i**); in **j** 50 μm (**j, j**″); in **k** 75 μm (**k, k**″). *CP* cortical plate, *IZ* intermediate zone, *LGE* lateral ganglionic eminence, *MGE* medial ganglionic eminence, *MZ* marginal zone, *PPL* preplate layer, *SP* subplate, *Str* striatum, *SVZ* subventricular zone

In the cortex, *Cdh13* is present in layers through which interneurons migrate, suggesting it may be expressed in these migrating cells. To confirm this, we carried out fluorescent in situ hybridization in GAD67-GFP mice, in which all interneurons express green fluorescent protein (Tamamaki et al. 2003). At E13.5, the staining appears to begin just underneath the PPL migratory stream and extends halfway towards the one at the level of the IZ (Fig. 2a–c, hollow arrowheads indicate the positions of migratory streams). By E15.5, *Cdh13* is strongly expressed in the CP, and delineates the region between the interneuron migratory streams of the SP and marginal zone (MZ) (Fig. 2d–f). At higher magnification, interneurons in the SP and CP do not express *Cdh13* mRNA (arrowheads in Fig. 2j, j″). At E18.5, expression of the cadherin has broadened, encompassing most cortical layers and appears to co-localize in some interneurons in the SVZ and IZ (open arrowheads in Fig. 2h) and when examined at higher magnification, in a few cells in the CP (open arrowheads in Fig. 2k, k″).

**Fig. 3:**
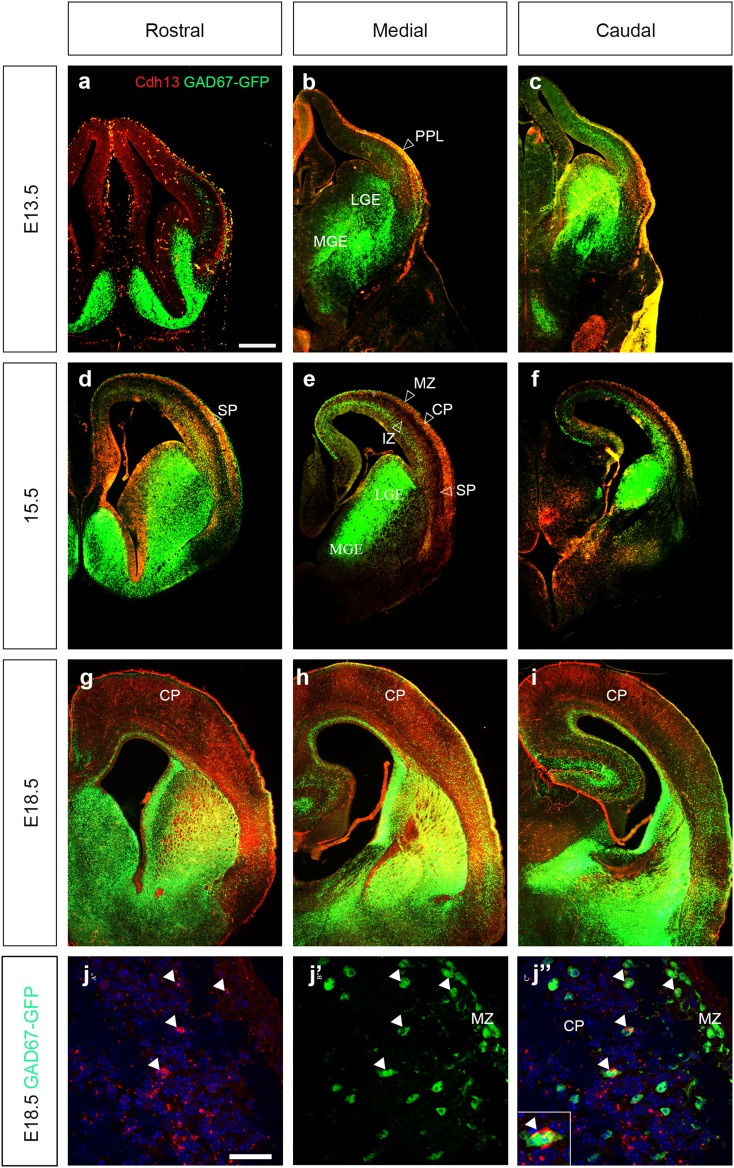
Expression of Cdh13 protein in cortical interneurons. Coronal sections from GAD67-GFP mouse forebrains at E13.5 (**a**–**c**), E15.5 (**d**–**f**) and E18.5 (**g**–**i**), were immunostained for Cdh13 (*red*) at rostral (**a, d, g**), medial (**b, e, h**) and caudal (**c, f, i**) levels. At E15.5 and E18.5, co-localization (*yellow*) is observed in the SP and CP. *Hollow arrowheads* indicate the positions of migratory streams of cortical interneurons. **j, j″** Higher power images at E18.5; *arrowheads* indicate examples of interneurons that express Cdh13 protein (see *inset*). *Scale bar* in **a** 500 μm (**a**–**i**), in **j** 75 μm (**j, j″**). *CP* cortical plate, *LGE* lateral ganglionic eminence, *MGE* medial ganglionic eminence, *MZ* marginal zone, *PPL* preplate layer, *SP* subplate

To confirm these findings, we carried out immunohistochemistry in GAD67-GFP mice for Cdh13 protein throughout the period of corticogenesis. At early to middle stages, the distribution of the protein mirrors that of the mRNA. At E13.5 there was strong yellow staining within the PPL, indicating co-localization (hollow arrowhead in Fig. 3a–c). However, on closer examination, this appears to be in blood vessels within the meninges (data not shown). At E15.5, there is strong staining in the CP, most likely in pyramidal neurons, and in the IZ concurrent with the in situ data (Fig. 3d–f); it is also present in the SP. Strong staining in the ventral telencephalon occurs only in the rostral portion and is seen in the germinal zones and the POA (Fig. 3d). At E18.5, Cdh13 protein expression pattern is strikingly different from the mRNA, where staining appears to be spread relatively evenly throughout all cortical layers (Fig. 3g–i). This ubiquity in staining is perhaps unsurprising, given the very broad *Cdh13* mRNA expression at this age. High-power confocal microscopy of sections at E18.5 shows punctate expression in a number of interneurons (see arrowheads Fig. 3j″), which is similar to that observed in human vascular smooth muscle cells (Philippova et al. 2003) and, more recently, in adult mouse hippocampal interneurons (Rivero et al. 2015). Our expression analysis throughout the period of corticogenesis has shown that Cdh13 is not expressed within proliferative zones in the developing forebrain, but is present within pyramidal neurons and some interneurons during mid-late stages of cortical development.

### Altered number of neurons in the cortex of *Cdh13*^−/−^ mice

**Fig. 4:**
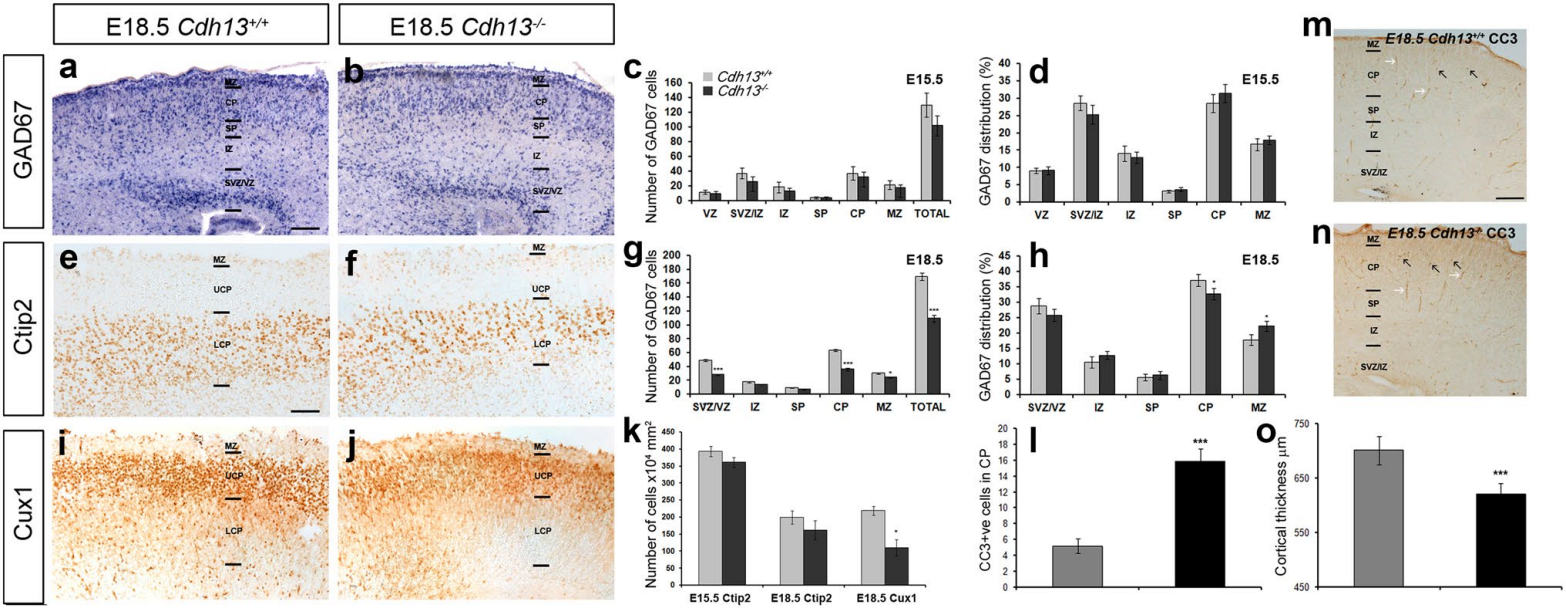
Reduced number of interneurons and pyramidal cells in the cortex of *Cdh13* ^−/−^ mice. Images of in situ hybridization for *GAD67* (**a, b**) and immunohistochemistry for Ctip2 (**e, f**), Cux1 (**i, j**) and CC3 (**m, n**) in coronal sections through the cortex of *Cdh13* ^+/+^ (**a, e, i, m**) and *Cdh13* ^−/−^ (**b, f, j, n**) mice at E18.5. Analysis of the number of *GAD67* labelled cells in all layers of the cortex at E15.5 (**c**) and E18.5 (**g**) as well as their relative distribution compared to wild-type litter mates at both ages (**d, h**). **k** Quantification of Ctip2 and Cux1^+^ cells in the cortex of *Cdh13* ^−/−^ animals showed reduced numbers of the latter only at E18.5 when compared to *Cdh13* ^+/+^ controls. **l** Quantification of the number of CC3+ cells in the CP (*black arrows* in **m, n**) showed an increase in the cortex of *Cdh13* ^−/−^ mice compared to *Cdh13* ^+/+^ controls at E18.5 [CC3+ blood vessels (*white arrows* in **m, n**, were excluded from counts)]. **o** Reduced cortical thickness was observed in the cortex of *Cdh13* ^−/−^ animals at E18.5 when compared to *Cdh13* ^+/+^ controls, to coincide with the decreased number of neurons. *Scale bars* in **a** 200 µm, in **e** 100 µm and in **m** 150 µm. (*p < 0.01, ***p < 0.0001). *Error bars* indicate SEM. *CP* cortical plate, *IZ* intermediate zone, *LCP* lower CP, *MZ* marginal zone, *SVZ* subventricular zone, *UCP* upper CP, *VZ* ventricular zone

Using in situ hybridization for *GAD67*, we assessed the number and position of interneurons within the developing cortex of *Cdh13* ^+/+^ and *Cdh13* ^−/−^ mice at middle (E15.5) and late (E18.5) stages of development (*n* = 5 for each genotype), at a time when expression of the gene was noted in these cell types. At E15.5, we did not observe any statistically significant differences in the total number of *GAD67*+ cells (*Cdh13* ^+/+^ 129.75 ± 16.4; *Cdh13* ^−/−^ 101.89 ± 13.3; *p* < 0.2) (Fig. 4c). These observations were confirmed using immunohistochemistry for the interneuron marker calbindin (CB) (Anderson et al. 1997) (data not shown). To assess potential positioning defects, we quantified the relative numbers of GAD67+ cells in each cortical layer as a percentage of the total number of neurons for each genotype. This analysis showed that loss of Cdh13 had no significant effect on their position within the cortex (Fig. 4d).

Analysis at a later phase of corticogenesis (E18.5) revealed a significant decrease in the total number of GAD67+ cells in the middle regions (along the rostral–caudal axis) of the cortex of (*Cdh13* ^+/+^ 169.56 ± 5.49; *Cdh13* ^−/−^ 109.37 ± 4.87; *p* < 0.0002), particularly in the CP (*Cdh13* ^+/+^ 63.03 ± 1.59; *Cdh13* ^−/−^ 35.78 ± 1.86; *p* < 0.0003), SVZ/IZ (*Cdh13* ^+/+^ 48.95 ± 1.37; *Cdh13* ^−/−^ 28.28 ± 0.94; *p* < 0.0009) and MZ (*Cdh13* ^+/+^ 30.21 ± 0.98; *Cdh13* ^−/−^ 24.42 ± 0.77; *p* < 0.02) (Fig. 4g). When we assessed position of GAD67+ cells within the developing cortex of *Cdh13* ^−/−^ mice, we observed a small but significant shift of cells from the CP (*Cdh13* ^+/+^ 37.17 ± 1.91; *Cdh13* ^−/−^ 32.71 ± 1.82; *p* < 0.04) to the MZ (*Cdh13* ^+/+^ 17.82 ± 1.78; *Cdh13* ^−/−^ 22.33 ± 1.59; *p* < 0.04) compared to *Cdh13* ^+/+^ littermates (Fig. 4h).

To assess whether this effect is specific to interneurons within the cortex, we immunostained coronal sections from *Cdh13* ^+/+^ mice and *Cdh13* ^−/−^ littermates for 2 pyramidal markers, Ctip2-[early born, lower layer (Arlotta et al. 2005)] and Cux1 (late born, upper layer Cubelos et al. 2010). At E15.5, we did not observe any statistically significant differences in the total number of Ctip2-positive cells in the cortex of *Cdh13* ^−/−^ mice (*Cdh13* ^+/+^ 393.57 ± 15.26; *Cdh13* ^−/−^ 361.42 ± 14.52; *p* < 0.34) nor at E18.5 (*Cdh13* ^+/+^ 199.09 ± 19.87; *Cdh13* ^−/−^ 161.2 ± 28.18; *p* < 0.57) (see Fig. 4e, f, k). However, at this later age, we found a marked decrease in the number of Cux1^+^ neurons (*Cdh13* ^+/+^ 218.54 ± 12.82; *Cdh13* ^−/−^ 110.25 ± 23.67; *p* < 0.01) (Fig. 4i–k). The reduction in the number of interneurons and pyramidal cells in the cortex of *Cdh13* ^−/−^ mice could be due to a number of factors, including defects in migration into the cortex, reduced proliferation and/or an increase in apoptosis or a combination of all three.

### Interneuron migration in the cortex of *Cdh13*^−/−^ mice

A previous study has shown a role for Cdh13 in cell migration. Specifically, transfection of CDH13 siRNA constructs into a human bladder carcinoma cell line significantly promoted the migration of these cells compared with controls (Lin et al. 2013). Based on this finding, we would predict increased migration and a greater number of interneurons in the developing cortex of *Cdh13* ^−/−^ mice. However, at E15.5, we did not see any significant changes in the number of interneurons in the cortex, which would suggest that their migration is probably unaffected in *Cdh13* ^−/−^ mice. This finding was confirmed in in vitro knockdown studies using immortalized gonadotropin-releasing hormone secreting (GnRH) neurons (GN11 cells), where we failed to observe an effect on migration, suggesting that Cdh13 is unlikely to play a direct role in neuronal migration (Fig. 7c).

**Fig. 5:**
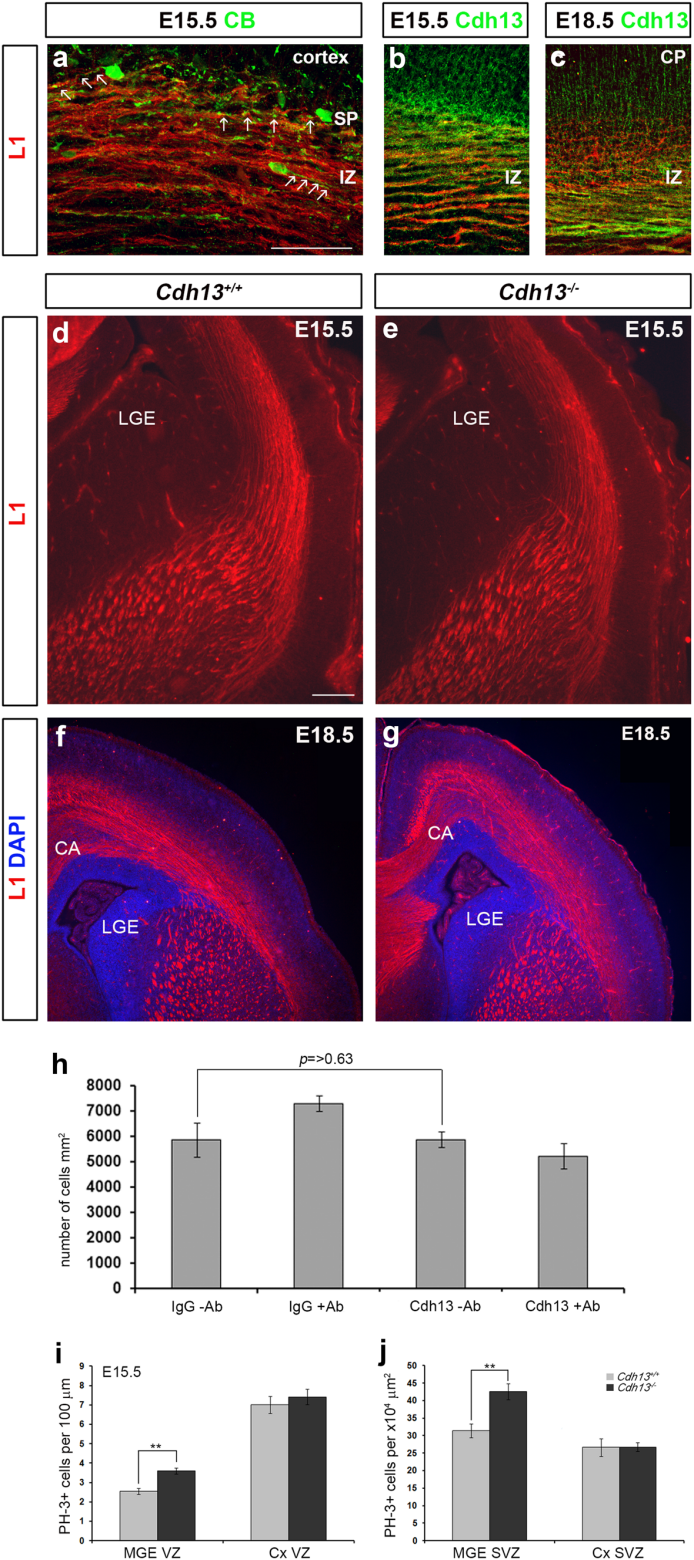
Normal axonal development in the cortex of *Cdh13* ^−/−^ mice. Coronal sections from wild-type mouse forebrains at E15.5 (**a, b**) and E18.5 (**c**) were immunostained for L1 (**a**–**c**), CB (**a**) or Cdh13 (**b, c**). At E15.5, interneuron processes appear to be in close contact with axons in the IZ (*arrows* in **a**) and, at E15.5 and E18.5, co-localization (*yellow*) is seen between L1 and Cdh13 in the IZ. Coronal brain sections from *Cdh13* ^+/+^ (**d, f**) and *Cdh13* ^−/−^ (**e, g**) mice at E15.5 (**d, e**) and E18.5 (**f, g**) were immunostained for L1. No gross differences were observed in axonal tract development. **h** Quantification of MGE cell migration in a Boyden’s chamber assay with membrane coated with human IgG or Cdh13Fc protein in the presence or absence of Cdh13 blocking antibody. Histogram shows coating membranes with Cdh13 protein or the addition of Cdh13 antibody does not affect interneuron migration. **i, j** Histograms indicate the presence of a significantly greater number of mitotically active (PH-3+) apical (VZ) and basal (SVZ) progenitors in the MGE of *Cdh13* ^−/−^mice at E15.5 compared to controls, but not in the cortex. *Scale bar* in **a** 100 µm, and in **d** 200 µm. Histograms show mean values and *error bars* represent SEM; **p < 0.001. *IZ* intermediate zone, *CP* cortical plate, *CA* callosal axons, *LGE* lateral ganglionic eminence, *SP* subplate

A number of studies have reported that interneurons use axonal projections to migrate from the ventral telencephalon to the cortex (Denaxa et al. 2001; McManus et al. 2004; Morante-Oria et al. 2003; Parnavelas 2000). While such an association was not supported by another study (Tanaka et al. 2003), knockdown of CXCL12 expression was recently shown to result in impaired axonal development and perturbed interneuron migration (Abe et al. 2015). These data suggest that axonal tracts in the cortex may be involved in interneuron migration after all. Interestingly, our expression analysis here seems to show that axonal tracts in the IZ express Cdh13 (Fig. 3e). To investigate an association between interneurons and axonal tracts within the developing forebrain and expression of Cdh13 in these axons, we carried out immunohistochemistry for CB, the thalamocortical axonal marker L1 and Cdh13. We found at E15.5, in agreement with previous studies (Parnavelas 2000; Denaxa et al. 2001), that many processes of migrating interneurons were in close association with axons in the IZ (arrows in Fig. 5a). Double labelling experiments for L1 and Cdh13 at E15.5 and E18.5 showed that thalamocortical axonal projections express both markers (Fig. 5b, c), suggesting that interneurons may use these Cdh13 expressing axons in their journey from the ventral to dorsal forebrain. Thus, any alterations in the thalamocortical and corticothalamic axonal projections may affect interneuron migration (Abe et al. 2015; Andrews et al. 2006; Denaxa et al. 2001) and, thus, explain the observed decrease in cortical interneurons in *Cdh13* ^−/−^ mice.

To assess the development of axonal tracts, we carried out immunohistochemistry for L1 at E15.5 and E18.5 in *Cdh13* ^+/+^ and *Cdh13* ^−/−^ littermates (*n* = 3 for each genotype). No gross differences were observed at either age in the development of axonal tracts (thalamocortical and corticothalamic) in *Cdh13* ^−/−^ compared to controls (Fig. 5d–g). However, as Cdh13-mediated de-adhesion can facilitate human aortic smooth muscle (SMC) and human umbilical vein endothelial (HUVEC) cell migration in a Boyden’s chamber assay (Ivanov et al. 2004b), we reasoned that Cdh13 expressed on axonal tracts could assist in the migration of interneurons. Consequently, loss of Cdh13 function would perturb the migration process and potentially explain why we see fewer cortical interneurons in the *Cdh13* ^−/−^ mice.

To test this idea, we mimicked interneuron migration along Cdh13 expressing axons in Boyden’s chamber assay experiments using E17.5 cortical interneurons and membranes coated either with Cdh13-Fc protein or human IgG, in the presence or absence of a Cdh13 blocking antibody. No significant differences were observed in the migration of interneurons on membranes coated with human IgG, compared to those coated with Cdh13-Fc protein (IgG 5857 ± 670 cells/mm^2^; Cdh13-Fc 5869 ± 309 cells/mm^2^, *p* > 0.63) (Fig. 5h). Addition of blocking antibody also failed to have a significant effect on migration, either on IgG or Cdh13-coated membranes compared to controls, suggesting that blocking Cdh13 function in interneurons and/or its substrate does not perturb migration. These findings are in agreement with our E15.5 cortical interneuron counts, where we failed to observe a change in interneuron numbers in the cortex, implying migration was unaffected by loss of Cdh13 function. Taken together, our data do not provide evidence for a role of Cdh13 in cortical interneuron migration.

### Altered proliferation in the developing forebrain of *Cdh13*^−/−^mice

A previous study demonstrated that expression of Cdh13 in neuroblastoma cells resulted in their inability to respond to epidermal growth factor-induced proliferation, indicating it functions as a negative regulator of neuronal cell growth (Takeuchi et al. 2000). Based on these in vitro observations, we would predict that loss of Cdh13 function would result in increased proliferation and more interneurons, not fewer, as observed here. To assess proliferation in these mice, we immunostained coronal sections from E15.5 *Cdh13* ^−/−^ mice and wild-type littermates (*n* = 3 for each genotype) for the mitotic marker PH-3. Analysis revealed a significant increase in the number of MGE PH-3+ cells in the VZ (*Cdh13* ^+/+^ 2.5 ± 0.161; *Cdh13* ^−/−^ 3.6 ± 0.16, per 100 µm; *p* < 0.00008) and SVZ (*Cdh13* ^+/+^ 31.45 ± 1.98; *Cdh13* ^−/−^ 42.6 ± 2.26 4 × 10^4^ µm^2^; *p* < 0.0009), but no changes in the cortical VZ (*Cdh13* ^+/+^ 7.01 ± 0.445; *Cdh13* ^−/−^ 7.42 ± 0.4 per 100 µm; *p* < 0.506) or SVZ (*Cdh13* ^+/+^ 26.64 ± 2.54; *Cdh13* ^−/−^ 26.75 ± 1.26 4 × 10^4^ µm^2^; *p* < 0.097) (Fig. 5i, j). While these data fit our prediction of increased number of proliferating progenitors in *Cdh13* ^−/−^ mice, they do not explain why we find fewer cortical interneurons at E18.5.

### Increased apoptosis in the developing forebrain of *Cdh13*^−/−^mice

A previous study demonstrated that up-regulation of Cdh13 protects endothelial cells from endoplasmic reticulum stress-induced apoptosis (Kyriakakis et al. 2010). Thus, here we reasoned that loss of Cdh13 function may lead to increased apoptosis. When we stained E18.5 sections for the apoptotic marker-cleaved caspase 3 (CC3), we did indeed observe a significant increase in the number of apoptotic cells in the CP of *Cdh13* ^−/−^ mice (*Cdh13* ^+/+^ 5.2 ± 0.92; *Cdh13* ^−/−^ 15.87 ± 1.53; *p* < 0.0004) (Fig. 4l–n). As a result of increased apoptosis and reduced neuronal numbers in the cortex of *Cdh13* ^−/−^ mice, we found a corresponding decrease in cortical thickness at E18.5 (*Cdh13* ^+/+^ 700.9 ± 25.97 µm; *Cdh13* ^−/−^ 620.72 ± 20.75 µm; *p* < 0.0001), but not at E15.5 (*Cdh13* ^+/+^ 498.57 ± 6.67 µm; *Cdh13* ^−/−^ 507.62 ± 7.66 µm; *p* < 0.64) (Fig. 4o). Taken together, our data indicate that loss of Cdh13 function results in increased apoptosis and a concomitant reduction in the number of Cux1^+^ neurons and cortical interneurons late in development.

### Loss of Cdh13 function affects apoptosis in neuronal cultures

**Fig. 6:**
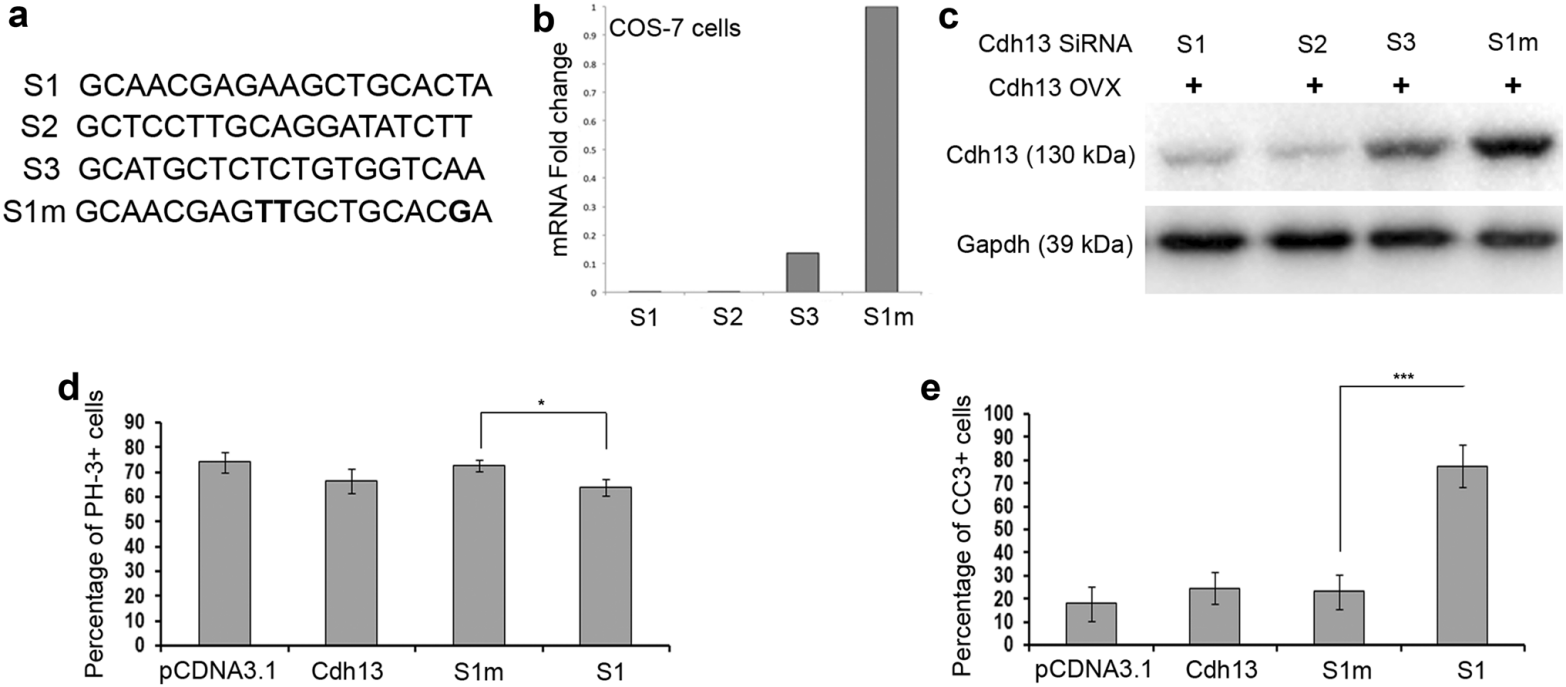
Suppression of Cdh13 expression increases apoptosis in interneurons. **a** Sequences of specific and triple-point-mutated (highlighted in *bold*) siRNAs targeting mouse Cdh13 mRNA. **b, c** COS-7 cells were transfected with Cdh13expression plasmid and different siRNA constructs. Two days after transfection, cell lysates were subjected to quantitative PCR (**b**) and Western blot analysis using Cdh13 antibody (**c**). Cdh13 levels in cells transfected with specific siRNAs (S1, S2) were significantly lower than those in cells transfected with triple-point-mutated siRNAs (S1m). Quantitation of proliferating (PH-3+) (**d**) and apoptotic (CC3+) (**e**) cells in E14.5 (**d**) and E17.5 (**e**) MGE cultures 2DIV after transfection with Cdh13 overexpression and knockdown constructs. Error bars indicate SEM (*p < 0.01, ***p < 0.0001)

To assess whether the defects observed in Cdh13 knockout mice is cell-autonomous, we decided to apply the RNAi technique to investigate the role(s) of this gene in corticogenesis. We designed three RNAi sequences (called S1–S3) that target three different regions within *Cdh13* mRNA (Fig. 6a). To test the effectiveness of our siRNA constructs on Cdh13 expression, we transfected COS-7 cells with the overexpression and either knockdown or mutated siRNA constructs (at a ratio of 1:3). 48 h after transfection, we assessed the level of Cdh13 mRNA and protein in these cells. We observed a significant reduction in both Cdh13 mRNA and protein with knockdown constructs, but not with mutated sequences (Fig. 6b, c).

To examine the effects of overexpression or silencing of Cdh13 on cell proliferation and apoptosis, Cdh13 cDNA or control (pCDNA3.1) and S1 siRNA(GFP) or mutated Sl siRNA(GFP) (S1m) were introduced into E14.5 rat MGE (equivalent to mouse E12.5) neural progenitors in order to examine proliferation or into E17.5 (equivalent to mouse E15.5) MGE cultures to assess apoptosis. After 2 days in vitro (2 DIV), cells were stained for nestin (neuronal progenitor) and PH-3 (proliferative) to assess proliferation or CB (interneuron) and CC3 (apoptotic) markers to identify apoptotic interneurons (see methods section for more details). Overexpression of Cdh13 did not have an effect on either proliferation (pCDNA3.1 73.98 ± 4.24% (EGFP/nestin/PH3 positive cells), Cdh13 66.43 ± 5.03%, *p* < 0.36) or apoptosis (pCDNA3.1 18.01 ± 7.43% (EGFP/CB/CC3 positive cells), Cdh13 24.6 ± 6.84%, *p* < 0.54) compared to control cultures (Fig. 6d, e). While Cdh13 knockdown had a small, but significant effect on proliferation within our neuronal progenitor cultures (S1m 72.64 ± 2.25% (EGFP/nestin/PH3 positive cells), S1 63.94 ± 3.5%, *p* < 0.017), it dramatically increased the level of apoptosis in our interneuron cultures (S1m 23.15 ± 7.5% (EGFP/CB/CC3 positive cells), S1 77.38 ± 9.25%, *p* < 0.0007) compared to controls, which is in agreement with our knockout mouse studies.

**Fig. 7:**
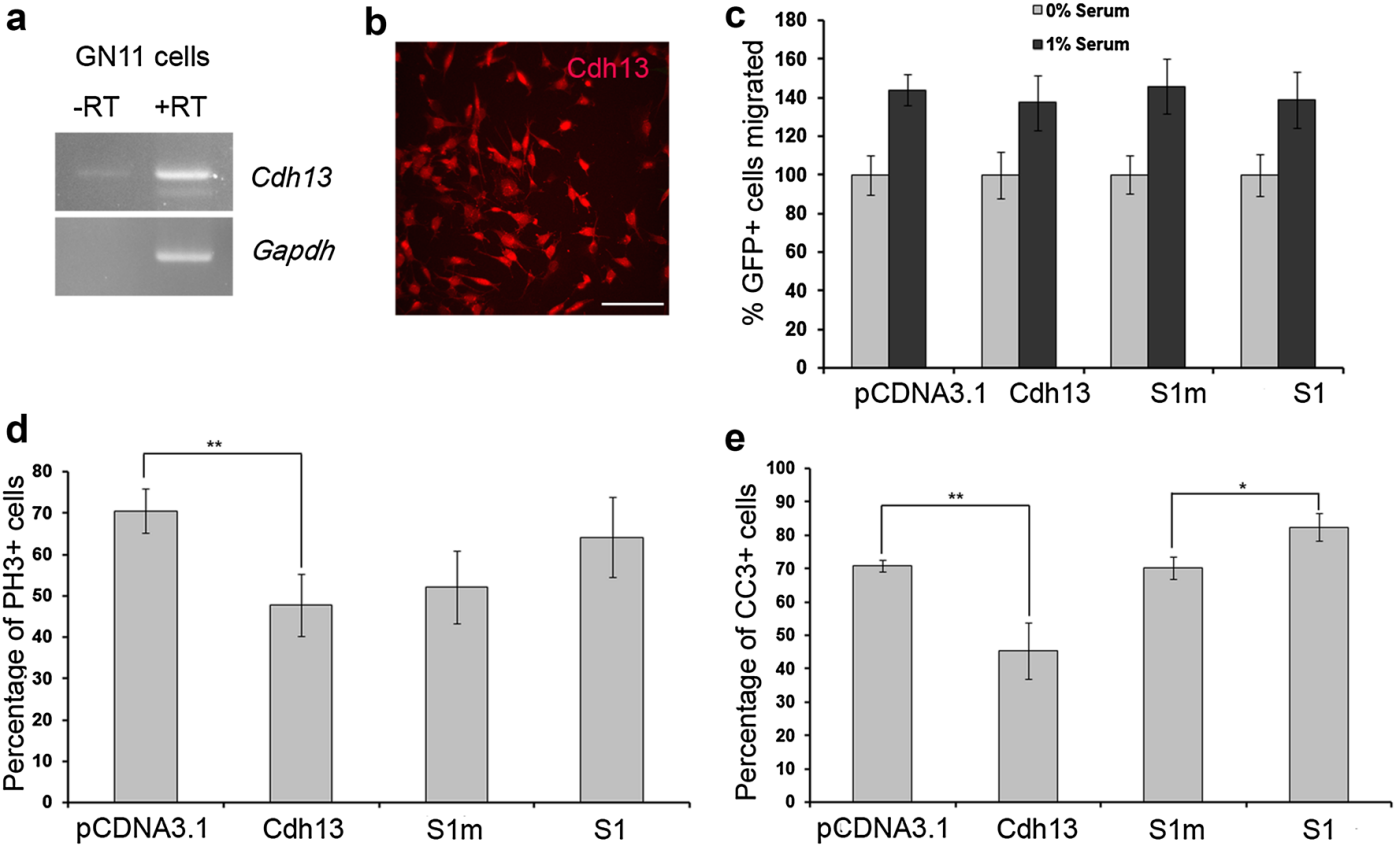
Suppression of Cdh13 expression in GN11 affects proliferation and apoptosis. **a** Reverse transcription (RT)-PCR analysis showed expression of Cdh13 in GN11 cells. **b** Immunohistochemistry confirmed the expression of Cdh13 receptor in GN11 cells. **c** Quantification of GN11 cell migration in a Boyden’s chamber following transfection with either Cdh13 overexpression, knockdown or control constructs showed that migration is unaffected by perturbing Cdh13 expression. Histograms show percentage of proliferating (GFP+/PH-3+) (**d**) and apoptotic (GFP+/CC3+) (**e**) cells following transfection with either overexpression, knockdown or control constructs. *Scale bar* in **b** 250 µm. (*p < 0.01, **p < 0.001). *Error bars* indicate SEM

To further characterize the putative role of Cdh13 in migration, proliferation and apoptosis, we wanted to determine whether the observed increase in cell death was specific to cortical interneurons or whether it applied to other migrating neuronal cell types. In earlier studies, we had used GN11 cells to examine the effects of factors on migration (Hernandez-Miranda et al. 2011) and on proliferation/apoptosis (Cariboni et al. 2011). Here, we explored whether GN11 cells also possess Cdh13 receptors and assessed the role of the cadherin on migration, proliferation and survival. We found that GN11 cells, like interneurons, expressed mRNA for Cdh13 by reverse transcription-PCR (Fig. 7a) and by immunohistochemistry (Fig. 7b). We next examined the effects of overexpression and knockdown of Cdh13 on GN11 cell migration using a Boyden’s chamber assay. Cells were cultured in the absence (negative control) or presence of 1% serum, which has a positive effect on migration (Cariboni et al. 2007). When we compared the percentage increase in migration following serum treatment on the different cell populations, we observed no significant differences in their migratory ability compared to controls (pCDNA3.1 144 ± 10.1%, Cdh13 ovx 137.49 ± 8.04%, *p* < 0.409; S1m 145.86 ± 14.1%, S1 139 ± 14.22%, *p* < 0.96) (Fig. 7c). In terms of proliferation, we observed a small but significant decrease in PH-3+ cells following overexpression, but not knockdown of Cdh13 levels (pCDNA3.1 70.57 ± 5.44%, Cdh13 ovx 47.74 ± 7.43%, *p* < 0.007; S1m 52.14 ± 8.85%, S1 64.18 ± 9.75%, *p* < 0.48) (Fig. 7d). These findings are in agreement with our E15.5 knockout mouse data, where we failed to observe any significant changes in the number of interneurons in the cortex or in their migration. However, when we examined the levels of apoptosis in our cultures following serum starvation, we observed overexpression of Cdh13 resulted in a dramatic decrease (pCDNA3.1 70.78 ± 3.34%, Cdh13 ovx 45.41 ± 8.5%, *p* < 0.002) and, conversely, knockdown increased the level of apoptosis (S1m 70.22 ± 3.28%, S1 82.39 ± 4.09%, *p* < 0.011) in GN11 cells (Fig. 7e), which is in agreement with our primary interneuron cultures.

### Cdh13 plays a protective role in neuronal development via the Pi3k/Akt pathway

**Fig. 8:**
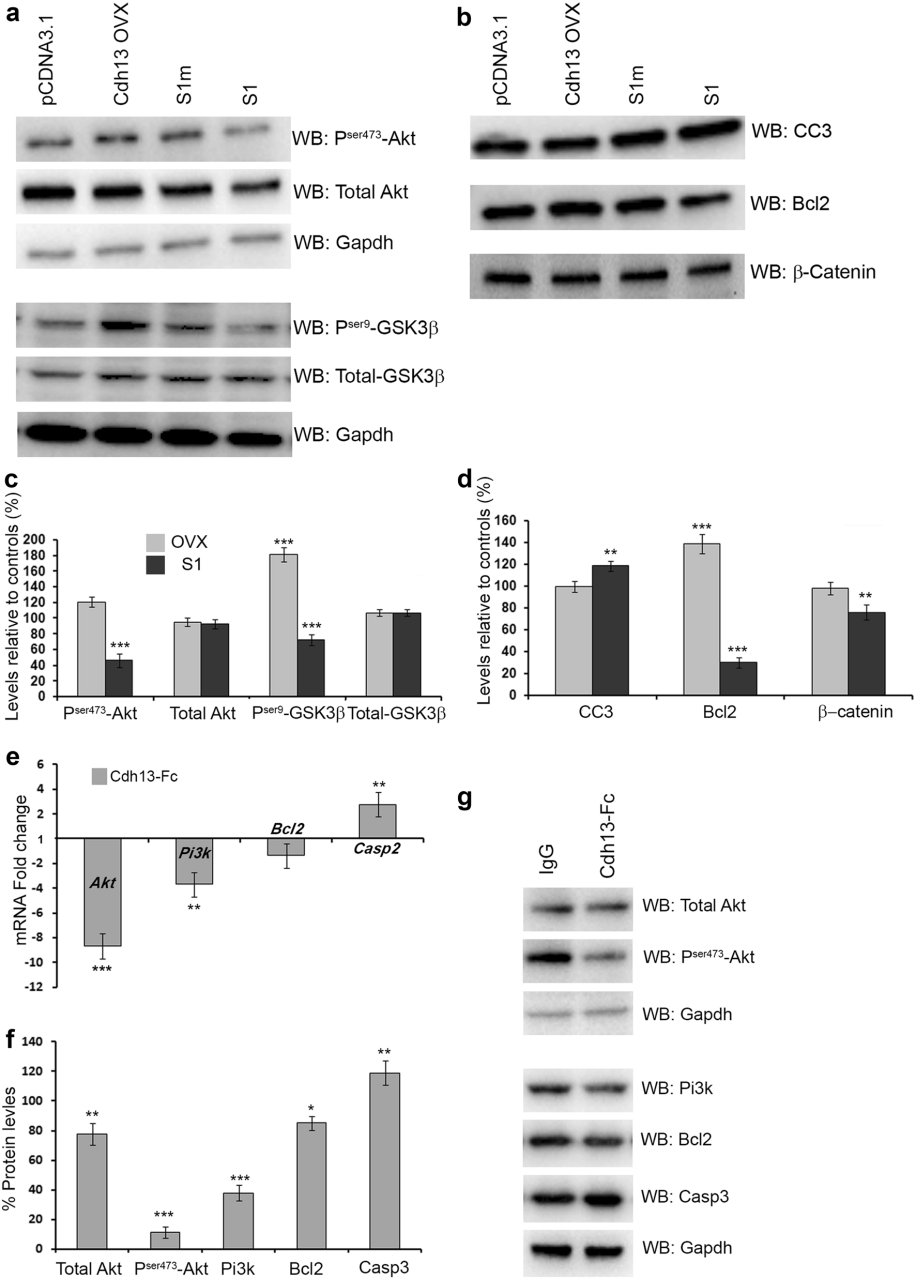
Increased apoptosis in GN11 cells and interneurons. Immunoblots of total and phosphorylated forms of Akt, GSK3β and Gapdh (**a**), and CC3, Bcl2 and β-Catenin (**b**) in GN11 cells following overexpression and loss of Cdh13. **c, d** Histograms show protein levels relative to controls, with a reduction in the levels of P^ser473^-Akt, P^ser9^-GSK3β and the anti-apoptotic marker Bcl2 and concomitant increase in the level of CC3 following Cdh13 knockdown. Changes in expression levels of mRNA (**e**) and protein (**f, g**) of genes involved in the Pi3k/Akt pathway in interneurons following incubation with a dominant negative form of Cdh13 (Cdh13-Fc) as compared to control human IgG treated cells (*p < 0.01, **p < 0.001, ***p < 0.0001). *Error bars* indicate SEM

Previously, it has been shown in endothelial cells that Cdh13 is up-regulated in response to oxidative stress, and adenovirus-mediated overexpression protects against oxidative stress-induced apoptosis (Joshi et al. 2005). This study also demonstrated that Cdh13-promoted survival was associated with concomitant activation of the Pi3-kinase/Akt survival signalling pathway. To assess if this pathway is affected in GN11 cells following overexpression and knockdown of Cdh13, we carried out Western blot analysis. We found that overexpression of Cdh13 increased the level of P^ser473^-Akt (pCDNA3.1 100 ± 8.8%, Cdh13 ovx120.76 ± 6.8%, *p* < 0.05) and P^ser9^-GSK3β (pCDNA3.1 100 ± 7.3%, Cdh13 ovx 181.35 ± 8.9%, *p* < 0.0001). Cdh13 knockdown had the opposite effect, resulting in a decrease in the levels of both proteins (P^ser473^-Akt S1m 100 ± 5.2%, S1 46.13 ± 8.2%, *p* < 0.0001; P^ser9^-GSKβ S1m 100 ± 8.4%, S172.23 ± 6.5%, *p* < 0.0001) compared to controls (Fig. 8a, c), suggesting that the survival pathway is deactivated following the loss of Cdh13 function.

Cdh13 was recently shown to function as a pro-apoptotic tumour suppressor that antagonises AKT/CREB/AP-1/FOX03a signalling in melanoma cells (Bosserhoff et al. 2014). To assess if this pathway was affected, we looked at the expression of downstream targets of FOX03a, the anti-apoptotic marker Bcl2 and the apoptotic marker CC3. Our Western blot analysis showed that the level of Bcl2 was reduced following Cdh13 knockdown (S1m 100 ± 4.9%, S1 30.19 ± 4.8%, *p* < 0.0001), while there was a concomitant increase in the level of CC3 (S1m 100 ± 5.2%, S1 118.51 ± 4.7%, *p* < 0.0001), which is in agreement with our previous observations (Fig. 8b, d).

Cdh13 overexpression has been shown to increase cell cycle progression and proliferation of HUVEC cells (Ivanov et al. 2004a), and ILK-GSK3β signalling via Cdh13 has been shown to modulate active (non-phosphorylated) β-catenin levels and downstream proliferation events in endothelial cells (Joshi et al. 2007). To assess further if proliferation was affected following Cdh13 knockdown in GN11 cells, we carried out Western blot analysis for active β-catenin. Active β-catenin levels were reduced following Cdh13 knockdown (S1m 100 ± 9.1%, S1 76.08 ± 7.2%, *p* < 0.001), which is in agreement with our PH-3 interneuron progenitor proliferation experiments (Fig. 6d).

To assess if these pathways were affected in interneurons following inactivation of Cdh13 function, we incubated E16.5 MGE interneuron cultures with either a dominant negative form of Cdh13 (Cdh13-Fc) or control human IgG protein (2 µg/m; each) for 2DIV, and then examined the levels of mRNA and protein. The mRNA levels of *Akt, Pi3k* and *Bcl2* were all significantly lower following treatment with the dominant negative form of Cdh13 (*Akt*, −8.68 ± 1.05, *p* < 0.0001; *Pi3k*, −3.69 ± 0.174, *p* < 0.001; *Bcl2*, −1.36 ± 0.12 fold change), while levels of *Casp2* were considerably higher (*Casp2*, 2.77 ± 0.69 fold change, *p* < 0.001) compared to control-treated interneuron cultures (Fig. 8e). Similar changes were observed in protein levels with reduced amounts of total Akt (77.45 ± 7.3%, *p* < 0.001), P^ser473^-Akt (11.2 ± 3.63, *p* < 0.0001), Pi3k (37.8 ± 5.3%, *p* < 0.0001) and Bcl2 (85.14 ± 4.7%, *p* < 0.01), and increased levels of Casp2 (118.97 ± 8.2%, *p* < 0.001) compared to control-treated cells (Fig. 8f, g). Taken together, the in vitro and knockout data suggest that Cdh13 plays a protective role in neuronal development by acting through the Pik3/Akt pathway.

## Discussion

The origins and migratory routes of cortical interneurons in rodents are now well documented. Tracing studies have confirmed that the majority of these cells arise in MGE in the ventral telencephalon and migrate in tangentially oriented streams to enter the cortex (Marin and Rubenstein 2003; Metin et al. 2006). Once in the cortex, they leave their migratory streams to assume positions in the CP where they assemble into functional circuits with their pyramidal counterparts, contributing to a precise balance of synaptic excitation and inhibition in this region. It has been suggested that disruption of this balance results in neurological conditions such as epilepsy, Parkinson’s disease (Cobos et al. 2005; Kumar and Buckmaster 2006; Mallet et al. 2006; Sloviter 1987) and attention deficit hyperactivity disorder (ADHD) (D’Agati et al. 2014; Purkayastha et al. 2015). Several studies have linked disruptions in CDH13 function with susceptibility to ADHD. In particular, a single nucleotide polymorphism in CDH13 has been linked with altered performance in certain memory tests in ADHD sufferers (Arias-Vasquez et al. 2011; Rivero et al. 2013). Recent studies have shown that RNAi suppression of Cdh13 function in lower layer cortical neurons leads to aberrant axonal projections (Hayano et al. 2014). Further, adult *Cdh13* ^−/−^ mice display deficits in learning and memory due to alterations in hippocampal neuron function, similar to those observed in ADHD patients (Rivero et al. 2015).

The neurological phenotypes observed in the adult *Cdh13* ^−/−^ mice reported by Rivero et al. (2015) could have arisen from defects in corticogenesis. Thus, to explore this idea further, we carried out a thorough examination of the expression pattern of Cdh13 and neuronal phenotype(s) in *Cdh13* ^−/−^ mice during cortical development. Our analysis showed that Cdh13 is not present in interneurons during the early phase (E13.5) of corticogenesis. This finding is in disagreement with our previous microarray study where we showed expression of Cdh13 in the PPL (Antypa et al. 2011). However, interneurons were not purified from the isolated PPL in this study, so it is possible that other cells or blood vessels, which appear to express Cdh13, could have been isolated together with interneurons, thus contaminating the sample.

During mid-stages of corticogenesis, Cdh13 is highly expressed in pyramidal neurons in the CP and along axons in the IZ, but not in interneurons. This is in agreement with our knockout data where we failed to detect any significant change in the number or positioning of interneurons or in early-born pyramidal cells. At E18.5, Cdh13 is expressed more strongly and broadly throughout the cortex, including pyramidal neurons in the CP and some interneurons. At this age we found fewer late-born pyramidal neurons and interneurons in the cortex of *Cdh13* ^−/−^ mice, especially in the CP, indicating that Cdh13 has a role to play in cortical development.

We hypothesized that the reduction in the number of both cortical cell types could be due to various factors including altered migration, proliferation or apoptosis. At E15.5, we did not observe any changes in the number of cortical interneurons in *Cdh13* ^−/−^ mice compared to controls or in the migration of GN11 cells following Cdh13 knockdown, implying that defects in migration were unlikely to be responsible for their altered number at E18.5. An effect on migration could be indirect, as several previous studies have shown that changes in thalamocortical and corticothalamic projections can affect interneuron migration (Abe et al. 2015; Andrews et al. 2006; Denaxa et al. 2001). However, our L1 staining failed to show any gross differences in axonal projections in mice lacking the cadherin, but more detailed DiI/DiA tracing studies are required to assess more accurately these projections and their possible role in interneuron migration. Moreover, results of the in vitro Boyden’s assay show that blocking Cdh13 function in interneurons or its presence in the underlying substrate does not affect their ability to migrate. Taken together, these findings imply that Cdh13 is unlikely to play a role in interneuron migration, and that the changes observed in the cortex of *Cdh13* ^−/−^ mice at E18.5 are likely due to effects on proliferation and/or apoptosis.

Previously, it was shown that overexpression of Cdh13 in HUVEC and human SMC cells resulted in increased proliferation (Ivanov et al. 2004a). Based on this finding, we predicted that Cdh13 knockdown would decrease proliferation, resulting in the generation of fewer interneurons, thus explaining the observed cortical phenotype. However, we failed to detect expression of Cdh13 in the proliferative zones during interneuron generation, suggesting that altered proliferation is unlikely the cause for the observed reduction in cortical interneuron numbers. This was confirmed in in vitro knockdown experiments using MGE progenitors or GN11 cells where we failed to detect an effect on proliferation. However, we did observe a small increase in proliferation in the proliferative zones of the MGE in vivo, but no changes in interneuron numbers in the cortex at E15.5. Since, loss of Cdh13 function does not appear to significantly affect either migration or proliferation; it raises the possibility of a role for this gene in apoptotic events.

Programmed cell death plays a profound role in brain development, including the formation of the cortex. It has been documented that during early postnatal stages, both excitatory and inhibitory neurons in the cortex undergo apoptosis (Thomaidou et al. 1997). Our finding of increased apoptosis in the cortex at E18.5 concurs with existing studies on Cdh13 function which suggest a role in the regulation of apoptosis through multiple pathways (Bosserhoff et al. 2014; Joshi et al. 2007, 2005; Philippova et al. 2008). The circulating, adipocyte-secreted hormone adiponectin (APN) has been shown to exert a protective effect in the heart and vasculature under stress conditions, through its association with Cdh13 and activation the AMPK pathway (Denzel et al. 2010; Parker-Duffen et al. 2013). In the model of cultured endothelial cells (EC), Kyriakakis et al. (2010) have shown that up-regulation of Cdh13 during endoplasmic reticulum (ER) stress attenuates the activation of the pro-apoptotic PERK branch of the unfolded protein response cascade and, thereby, protect EC from ER stress-induced apoptosis. Thus, it is conceivable that signalling pathways similar to those activated by APN and/or the UPR play a role in Cdh13 apoptotic events within the developing cortex. Further studies are required to evaluate these possibilities.

**Fig. 9:**
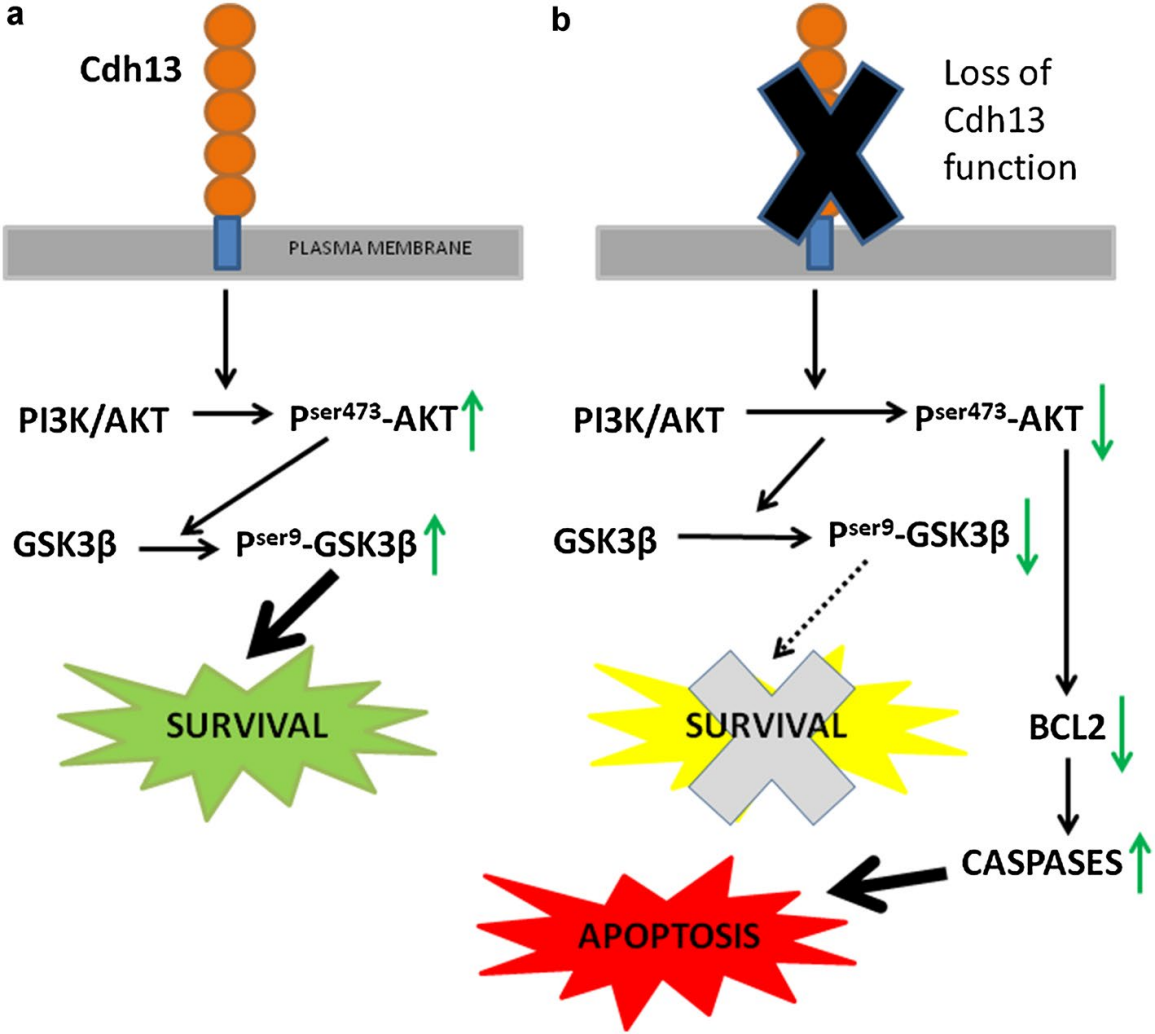
Schematic model for Cdh13-induced signal transduction. **a** Following activation of Cdh13, inwardly directed signals are mediated via intracellular effectors Akt/GSK3β which modulate subsequent downstream survival responses. **b** In the absence of Cdh13, levels of “active” phosphorylated Akt/GSK3β are reduced. This not only leads to inactivation of the survival pathway (*dashed line*), but also to reduced levels of the anti-apoptotic gene Bcl2 and the activation of the apoptotic pathway, thus leading to neuron death

A recent study by Southwell and colleagues found that 40% of cortical interneurons were eliminated through Bax- (Bcl-2 associated X-) dependent apoptosis in postnatal life (Southwell et al. 2012). Interestingly, the proportion of interneurons lost in the *Cdh13* ^−/−^ cortex (~36%) is broadly similar to that reported by these authors, suggesting a precocious occurrence of these events. Such a role is likely, as a recent study has shown that induced expression of Cdh13 in melanoma cells resulted in the detection of V-Akt and FoxO3a hypophosphorylation, which was accompanied by the down-regulation of the ant-apoptotic molecules BCL-2 and BCL-x, suggesting that Cdh13 regulates Akt signalling and desensitizes melanoma cells to apoptosis (Bosserhoff et al. 2014). Such a protective role was confirmed in in vitro studies, where we found altered levels of Akt/GSKβ signalling and reduced levels of Bcl2 and increased apoptosis following Cdh13 knockdown (Fig. 9). Thus, it is likely that Cdh13 signalling via Akt/GSKβ and Bcl-2 plays an important part in regulating the intrinsic timing mechanisms involved in interneuron cell death and survival.

In summary, we report here that the majority of cortical interneurons and pyramidal cells express Cdh13 at late stages of corticogenesis. Absence of this receptor leads to prenatal reduction in the number of both neuronal cell types. This decrease does not appear to be due to defects in migration or neurogenesis, but rather to increased apoptosis. These results implicate Cdh13 in the regulation of programmed cell death and survival in the developing forebrain that is prerequisite for the assembly of functional neuronal circuits.
